# Dentition and Its Derangements

**Published:** 1863-01

**Authors:** A. Jacobi


### Selections.

Dentition and its Derangements—*By A. Jacobi*, M. D. *LECTURE IX.*—Part 2.—Now, the principle results of these carefully collected and compared figures are, among others, these: The anterior lobes of the large hemispheres are small in proportion to the bulk of the brain. The cerebellum also is small in proportion to the cerebrum. Both the anterior lobes of the large hemispheres and the cerebellum grow more rapidly in early infancy than the generally rapid development of the infantile brain would explain. That the brain itself grows most, at the same period, in proportion to the other organs of the body, I have often had the opportunity of telling you. Finally, there is a little cortical, grey cerebral substance, and not the distinct difference between the grey and white substances of later life.

Just draw your physiological conclusions from these facts, translate as it were anatomy to physiology. The simplest physiological facts you would conclude from the data I have given, are these. The mental, intellectual faculties of the infantile brain are little developed; the less the earlier the period of our observation. The power over the voluntary muscles is very limited, indeed. The mutual counterbalancing of the really central, and the conducting portions of the brain is not well pronounced. There is, with the intense growth, intense action of the brain, in its course of development. The less settled the condition of an organ, the more it is exposed to irregular action. Therefore the great irritability of the brain in general is well explained by its anatomy; but particularly the intense irritability of the anterior lobes of the large hemispheres and the cerebellum.

This irritability shows itself less by increased physiological action, than by its irregularity and abnormity. For instance, the irritation of the sensory organs, the functions of which are transferred to the grey substance, does not increase their exactness and accuracy; that is, such patients do not see, hear and feel better, but they are molested by impressions which were hitherto normal and agreeable. There is intolerance to noise, scintillation, pain in the eye, and on the periphery of the cutaneous nerves.

The increased growth of the cerebral substance explains the great afflux of arterial blood with its normal amount of oxygen. This is particularly the case in those portions of the brain mentioned by me as the seats of intellectual and voluntary motory power. This physiological injection, however necessary for the normal development of the parts, becomes, by its very intensity, easily pathological, that is the transition from the healthy condition of the organ to disease is very easy indeed. You know that I have often pointed out to you the necessity of not looking on disease in general as something essential and peculiar in itself, but as the expression of the physiological functions and appearance under altered circumstances.

In the majority of cases of pathological cerebral hyperæmia, brain, cerebral membranes and integuments, are equally affected. Sometimes, however, the injection is noticed only in the larger, and also the smaller, bloodvessels of the membrane; very rarely the braina lone, and exclusively, is found to be the seat of the anomaly. In other cases the process is but a partial one in other respects; there are some in which only the white substance, or the cerebral portion of the brain, or a single hemisphere, or the cerebellum, or even the cortical substance alone are affected. Some of these facts may get nearer their explanation by their anatomical condition; perhaps the falx cerebri, and the cerebellum separating principal parts of the brain, prevent the uniform spreading of originally local hyperæmia. Discolorations of the arachnoid membrane, œdematous effusions of the pia mater, and small extravasations are frequent; œdematous mollification of the cerebral tissue, however, are rare companions of the above alteration.

There is no age in which cerebral hyperæmia is so frequent as early infancy, and therefore, also, the period of the first dentition. The newly-born and the healthy mal} nursling are particularly subject to the primary and idiopathic form, which often depends not only on the physiological afflux of a large amount of blood, but frequently also on the injurious effects of too high a temperature, tea and coffee, opium and alcohol. The secondary, consecutive form depends on disturbed circulation of the blood, in diseases of the lungs and heart, in overloaded stomach and intestine, inflamed peritoneum; in the somewhat advanced period of infantile life, acute exanthems, typhoid and other fevers, impaired condition of the blood, mental emotions, are not at all rare causes of cerebral hyperæmia. Concussion of the head, extensive and thorough refrigeration of the skin, are further occasional sources of the same affection, and attacks of convulsions produced by whatever cause, become dangerous by the secondary injection brought on by the spasmodic action of the muscles of the neck, and chest, and diaphragm, and abdomen, in a great many cases.

Among the symptoms of cerebral hyperæmia in children those of the motory organs are prevailing over those of the sensory ones. They are often mistaken for those of meningitis, and sometimes so easily that a correct differential diagnosis of the two is by no means possible, except on careful observation of the course of the disease. Generally, such children have suffered from costiveness, for several days, their sleep was restless, they ground their teeth, had frightening dreams, headaches perceptible by the perpendicular wrinkling of the corrugator muscles of the eye-brows, and took a long time before falling asleep. Vomiting will then set in, the pupil becomes contracted from the irritatien of the oculo motory nerve, and some muscles will be observed to suffer from spasmodic affections. These local twitchings are followed by general convulsions, or the case may be severe enough to exclude the gradual development of these symptoms. In such cases the convulsions, commencing in one limb, or in one side of the face, spread rapidly over the whole body. These general clonic convulsions sometimes alternate with tonic ones, general tetanus taking place; then again the muscles of the neck will be found rigid, in tonic contraction, while the clonic convulsions spread over all the muscles of the extremities. Sometimes a remission will take place after half an hour, or an hour, and the attack may stop altogether; but very frequently the former vehemence of the affection returns, and may even result in death. During the attacks there is no mental nor sensory reaction. The powerful irritations of the skin are not felt, the pupils do not react, consciousness is entirely gone. The skin is in perpetual perspiration, respiration is interfered with, saliva becomes foamy from the constant motions of the masticatory muscles, and the abdomen tympanitic. Nevertheless in almost every case the prognosis is favorable; always provided that meningitis, both the genuine and the tubercular forms, can be excluded. At the period of life of which I principally speak, both forms are not very frequent. Genuine, often so-called suppurative, meningitis is generally found after traumatic injuries of the head, or diseases of the cranium. Tubercular meningitis is rarely found before the first, or even second, year of life, and generally preceded by a number of long continued symptoms, of which I need not speak at this occasion.

The symptoms of gradually developed anæmia of the brain are very similar to those attending hyperæmia, as irritation sets in first, followed by depression and paralysis. These symptoms can not be well explained by a change in the pressure of the cerebral substance, which may be assumed to exist in hyperæmia, but it appears that a certain amount of pressure, resistance, or tension is necessary, and that both, too little and too much, are equally injurious. Moreover, physiological experiments have proved that there is always a stage of irritation preceding the entire extinction of nervous action; therefore, increased irritation is not the result of increased, but of diminished nutrition. Therefore, also, a moderate degree of cerebral anæmia is attended with the symptoms of irritation, the highest degrees, however, with those of paralysis. Thus Marshall Hall distinguishes an irritable and a torpid stage of hydrencephaloid disease. The first name is given to such cases of cerebral anæmia in children, in whom there is still irritation of the motory functions; the second is applied to those already paralysed. In the former, the children are restless, grind their teeth, show frequent symptoms of sudden fright, and shrill out in their sleep, and have a flushed face, frequent pulse, and increased temperature. At last, sometimes convulsions will be observed. The second stage, which will always follow a mistake made in either diagnosis or treatment, exhibits collapse and apathy in the patients; the power of accomodation is disturbed, the eyelids are half-closed, and the pupils show no reaction to the influence of light, respiration becomes irregular, and under pulmonary œdema and coma life becomes extinct.

I have deemed it proper to compare these two conditions, anæmia and hyperæmia, for their practical importance. Both are as frequent as they can become dangerous. You see, however, at once, from the various, both physiological and extraneous causes capable of producing hyperæmia of the brain, how little space is left for the assumption of dentition, that is the protrusion of a tooth, being in itself a cause of cerebral hyperæmia. The fact is, as I have often said, that hyperæmia of the brain, and such either local or general symptoms as are generally attributed to dentition, are coordinate results of the physiological process of local irritation and development, be they normal or excessive; with the exception of those few cases, were a local inflammatory process in the jaws or gums will give rise to fevers and their sequelæ.—*Medical Times.*

